# Pushing the temporal resolution in absorption and Zernike phase contrast nanotomography: enabling fast *in situ* experiments

**DOI:** 10.1107/S1600577520007407

**Published:** 2020-07-30

**Authors:** Silja Flenner, Malte Storm, Adam Kubec, Elena Longo, Florian Döring, Daniël M. Pelt, Christian David, Martin Müller, Imke Greving

**Affiliations:** a Institute of Materials Research, Helmholtz-Zentrum Geesthacht, Max-Planck-Strasse 1, 21502 Geesthacht, Germany; b Diamond Light Source Ltd, Didcot, Oxfordshire OX11 0DE, United Kingdom; c Paul Scherrer Institut, Forschnungsstrasse 111, 5232 Villingen, Switzerland; d Centrum Wiskunde and Informatica, Science Park 123, 1098 XG Amsterdam, The Netherlands

**Keywords:** nanotomography, full-field X-ray microscopy, *in situ* experiments, image quality, time resolution, Zernike phase contrast

## Abstract

Hard X-ray nanotomography enables 3D investigations of a wide range of samples with high resolution (<100 nm) with both synchrotron-based and laboratory-based setups. However, the advantage of synchrotron-based setups is the high flux, enabling time resolution, which cannot be achieved at laboratory sources. Here, the nanotomography setup at the imaging beamline P05 at PETRA III is presented, which offers high time resolution not only in absorption but for the first time also in Zernike phase contrast. Two test samples are used to evaluate the image quality in both contrast modalities based on the quantitative analysis of contrast-to-noise ratio (CNR) and spatial resolution. High-quality scans can be recorded in 15 min and fast scans down to 3 min are also possible without significant loss of image quality. At scan times well below 3 min, the CNR values decrease significantly and classical image-filtering techniques reach their limitation. A machine-learning approach shows promising results, enabling acquisition of a full tomography in only 6 s. Overall, the transmission X-ray microscopy instrument offers high temporal resolution in absorption and Zernike phase contrast, enabling *in situ* experiments at the beamline.

## Abstract 2

Unique transmission X-ray microscopy geometry allows high temporal resolution in absorption as well as phase contrast nanotomography. The evaluation of fast scan times versus image quality is presented.

## 1. Introduction   

Nanotomography is a widely used tool for 3D evaluation in materials science, for biological as well as medical sample systems. Using synchrotron radiation, full-field transmission X-ray microscopy (TXM) tomograms with high spatial resolution (<100 nm) are routinely recorded in time frames of 15 min to 1 h (Andrews *et al.*, 2010 ▸; Yuan *et al.*, 2018 ▸). Benchtop devices are evolving rapidly in terms of resolution, scanning time and image quality (Patterson *et al.*, 2016 ▸). TXMs have been implemented in benchtop machines and even phase contrast modalities are available, *e.g.* Zernike phase contrast (Zernike, 1934 ▸; Schmahl *et al.*, 1994 ▸). However, one major drawback of benchtop machines will not be resolved easily: the flux density at the sample is limited and therefore the time resolution cannot compete with synchrotron-based systems. Nanotomography setups at synchrotrons can offer fast scanning times and/or high image quality as well as phase-contrast modes thanks to the highly brilliant source. Recently, the first fast TXM experiments in absorption mode with scanning times of 1 min were reported at NSLS II using a highly efficient capillary condenser (Ge *et al.*, 2018 ▸). However, phase contrast methods such as Zernike phase contrast are currently much slower, since usually less efficient optics are used [beamshaping condenser, Koehler-like illumination (Vogt *et al.*, 2006 ▸)], whereas absorption contrast imaging can be used in combination with highly efficient capillary condensers. (Ge *et al.*, 2018 ▸) Other full-field techniques like holotomography have the potential to be very fast (Villanova *et al.*, 2017 ▸) but often require at least three distances to reconstruct specimen of arbitrary composition, which limits possible *in situ* application.

Improvements in X-ray optics [*e.g.* higher aspect ratio of zone plates, blazed zone plates (Mohacsi *et al.*, 2014 ▸)] and detectors and higher flux at new generation sources will reduce acquisition times for full-field nanotomography even further. There is, however, always a trade-off between scan time and contrast-to-noise ratio (CNR). The image quality strongly depends on the count rate at the detector and therefore on the exposure time (Waske *et al.*, 2010 ▸). Shorter exposure times reduce the image quality but allow for a higher sample throughput, reduction of sample movement caused by environmental factors and by long-term drifts, and a dose reduction, the latter being especially important for biological samples.

Here, we present a hard X-ray nanotomography setup based at a third-generation source, which offers high temporal resolution, not only for absorption but also for phase contrast methods. Tomographic scan times down to 6 s were achieved and the advantages and disadvantages of different scanning times are compared. The standard X-ray absorption microscopy as well as Zernike phase contrast are evaluated in terms of contrast and spatial resolution. The high-*Z* material nanoporous gold (NPG) was chosen as a test case for the absorption contrast tomography. This material has been proven to be well suited as a test object for evaluating TXM performance before (Larsson *et al.*, 2019 ▸). For the Zernike phase contrast we chose a low-*Z* phase object with nano-sized grains, namely a magnesium alloy (Ghasemi *et al.*, 2018 ▸; Penther *et al.*, 2018 ▸).

## 2. Materials and methods   

### 2.1. X-ray microscopy setup   

**Figure 1:**
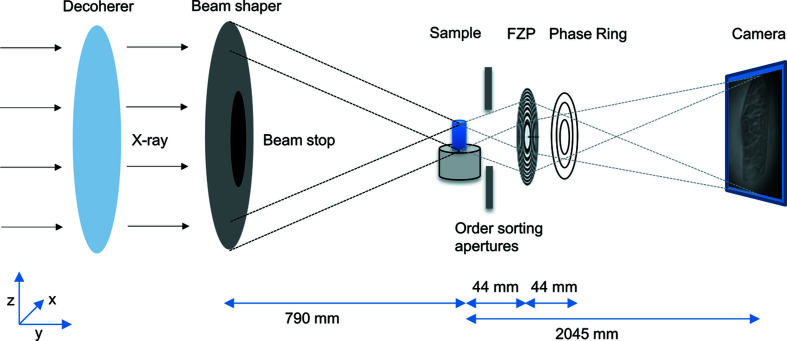
Schematic of the Zernike phase contrast setup at the imaging beamline P05.

The experiment was performed at the nanotomography endstation at the imaging beamline P05 operated by the Helmholtz-Zentrum Geesthacht at the PETRA III storage ring at DESY, where a full-field X-ray microscope has been installed (Ogurreck *et al.*, 2013 ▸; Greving *et al.*, 2017 ▸, 2018 ▸; Flenner *et al.*, 2018 ▸). A schematic of the setup is displayed in Fig. 1 ▸.

The beamshaping condenser (Jefimovs *et al.*, 2008 ▸; Vartiainen *et al.*, 2015 ▸) has a diameter of 1.8 mm with 50 nm finest structure size. Structures are made with HSQ (hydrogen silsesquioxane) resist on an Si_3_N_4_ membrane with an Ir ALD (atomic layer deposition) coating (Vila-Comamala *et al.*, 2011 ▸), providing an illumination at the sample plane of 50 µm × 50 µm. A Fresnel zone plate (FZP) made from gold on an Si_3_N_4_ membrane of 250 nm thickness with outermost zone width *dr* = 50 nm and a diameter of 100 µm (*i.e.* number of zones *N* = 500) is used as an objective lens (Gorelick *et al.*, 2011 ▸), resulting in a focal distance of 44 mm at 11 keV. The Zernike phase rings are fabricated from gold on an Si_3_N_4_ membrane of 250 nm thickness and the structure height of ∼1.1 µm was chosen to give a phase shift of π/2 at an energy of 11 keV. Phase rings with line widths between 0.5 µm and 1.3 µm were used. All optics were designed and manufactured at the Paul Scherrer Institut. To reduce coherence effects from the source, a rotating paper (standard printing paper) is used as a decoherer.

An X-ray sCMOS camera (Hamamatsu C12849-101U, 6.5 µm pixel size, 2048 × 2048 pixels, 16 bit image depth) with a 10 µm Gadox scintillator was used as a detector. The scintillation layer is directly coupled to the sCMOS chip and results in a high photon efficiency. The detector was placed 20.45 m behind the sample. Because of this large sample-to-detector distance possible at this instrument, no light optical magnification is necessary, enabling a high photon efficiency. With this setup, a pixel size of down to 13 nm and a spatial resolution of 50 nm have been achieved at 11 keV in 2D. In addition to standard absorption microscopy, Zernike phase contrast can be performed by adding phase rings in the back focal plane of the FZP. During the tomographic scan, the sample was rotated continuously at a constant speed (fly scan mode) with a high-precision air-bearing rotation axis (PI miCos custom design; the motion errors are given in Table S1 of the supporting information). This reduces the total scanning time and eliminates sample movements induced by the repetitive acceleration and deceleration.

However, a continuous rotation has a drawback: the sample will move during each image. As the rotation is an angular motion, the absolute linear motion in the projection is largest for the outermost parts of the sample. If this motion is limited to a value of *b* pixels (with *b* ≤ 1 for sub-pixel blurring), the exposure time *t*_exp_ per projection and the total scan time *t*_scan_ (which is determined by the rotation speed and therefore defines how fast the sample moves) are linked by the following formula,
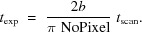
The number of images acquired in one scan does not only depend on the exposure time and rotation speed but also on the overhead required by the camera. The Hamamatsu camera can bin the image directly on the chip, therefore the overhead time (camera readout time) with the current infrastructure can be reduced from 0.1 s (no binning) to 0.05 s (binning 2) and 0.04 s (binning 4). The binning and the number of images influence the ratio between the total exposure time *t* and the total scan time *t*_scan_ (efficiency *E* = *t*/*t*_scan_). A higher binning increases the signal-to-noise ratio, reduces the overhead time (higher *E*) and allows for a longer exposure time without blurring (because of fewer pixels), and therefore increases the efficiency.

Scans were performed with total scan times from 53 min (absorption) and 15 min (Zernike phase contrast) down to 6 s. In total, we performed nine scans for absorption and six scans for Zernike phase contrast with different parameters (*e.g.* exposure time, number of projections, binning), which can be found in Table 1 ▸. For the very short scans of 18 s and 6 s, a maximum shift *b* was allowed to be larger than one pixel in order to keep a reasonably high efficiency, *i.e.* keeping the total dead-time of the detector as small as possible. Since the detector has a point spread function of 2.5 pixels, this is acceptable.

### 2.2. Reconstruction and analysis   

All reconstructions were performed with the *Gridrec* algorithm (Dowd *et al.*, 1999 ▸) and a Shepp–Logan filter using the *TomoPy* package (Gürsoy *et al.*, 2014 ▸). The observed drifts of up to 500 nm during 1 h in the vertical direction can be easily corrected for (Storm *et al.*, 2017 ▸; Guizar-Sicairos *et al.*, 2011 ▸), which leads to significant reduction in ring artifacts (Pelt & Parkinson, 2018 ▸). However, the movements in the *x* direction in the range of 200 nm during 1 h are much more difficult to correct for. Several alignment methods are available for absorption contrast, but most of them fail for Zernike phase contrast because of artifacts induced by the phase ring. Drift correction in the vertical direction was performed for the 15 min scans, usually in the range of 3–4 pixels (unbinned). For shorter scans, this is not necessary. The segmentation of the reconstructed volumes was carried out by automated thresholding based on the minimum method (Prewitt & Mendelsohn, 1966 ▸). For the fast scans, filters were applied and compared. A fast Fourier transform bandpass filter (low pass) and a median 3D filter with a σ of 2 pixels turned out to be most efficient while keeping edges for the shortest scan times (van der Walt *et al.*, 2014 ▸). For longer scan times, a median 3D filter in combination with a non-local means filter gave the best results. The CNR was calculated by the following equation (Muhogora *et al.*, 2008 ▸), written for gold and air, for example,
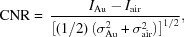
where *I*_Au_ and *I*_air_ are the mean grey values in gold and air, and σ_Au_ and σ_air_ are the standard deviations for these materials. The ratio is calculated on the unfiltered data using the mask of the segmented volume. All data were binned to the same effective pixel size before reconstruction to eliminate the influence of binning on the CNR. Since the magnesium sample consists of two different materials (SiC particles and Mg matrix) and voids, the CNR between each material and the voids, as well as the CNR between these two materials, can be calculated.

The resolution of the 2D/3D volume was estimated via Fourier ring/shell correlation (FRC, FSC). In the 3D case, the projections were divided into two stacks and the two reconstructed volumes were then used to estimate the FSC. Here, the original binning level for each scan was used (see Table 1 ▸). We used the half-bit threshold criterion to calculate the resolution from the FRC/FSC curves (van Heel & Schatz, 2005 ▸).

### 2.3. Materials   

NPG has recently received increasing interest (Lilleodden & Voorhees, 2018 ▸; Weissmüller & Sieradzki, 2018 ▸; Qi & Weissmüller, 2013 ▸) and is an ideal test sample for the characterization of nanotomography setups. The size of the gold ligaments can be tailored depending on the expected resolution of the setup. The ligament size in the sample used in this experiment is 250 nm (Larsson *et al.*, 2019 ▸). Gold is a strongly absorbing material at 11 keV, with a δ/β of only 6.25 (optical material parameter given in Table S2). The calculated transmittance of a sample of 10 µm diameter is 18% at 11 keV, so a good contrast in absorption mode is expected.

As a test sample for Zernike phase contrast, we used a low-absorbing magnesium composite with 10 vol% SiC particles of sub-micrometre size (Penther *et al.*, 2018 ▸; Ghasemi *et al.*, 2018 ▸). This allowed testing the resolution by analyzing larger particles and smaller particles. The absorption of magnesium, as well as SiC, is very low. The calculated absorption for a sample of 20 µm diameter is only 5.7% at 11 keV and the δ/β ratios are >100 for Mg and SiC. Therefore, this sample is well suited as a test sample for Zernike phase contrast.

The magnesium composite and NPG sample do have different natures: the NPG sample has a binary structure (gold–air) which yields hard boundaries with many high-frequency contributions, while the magnesium composite has fewer very fine structures and the contrast between its main constituents (SiC grains and Mg) is weaker than between gold and air. Therefore, a direct comparison regarding the spatial resolution and CNR for these two samples is misleading and should not be made. Choosing these different samples, however, offers a guideline for a wide range of samples.

## 3. Results   

### 3.1. Standard X-ray absorption microscope   

**Figure 2:**
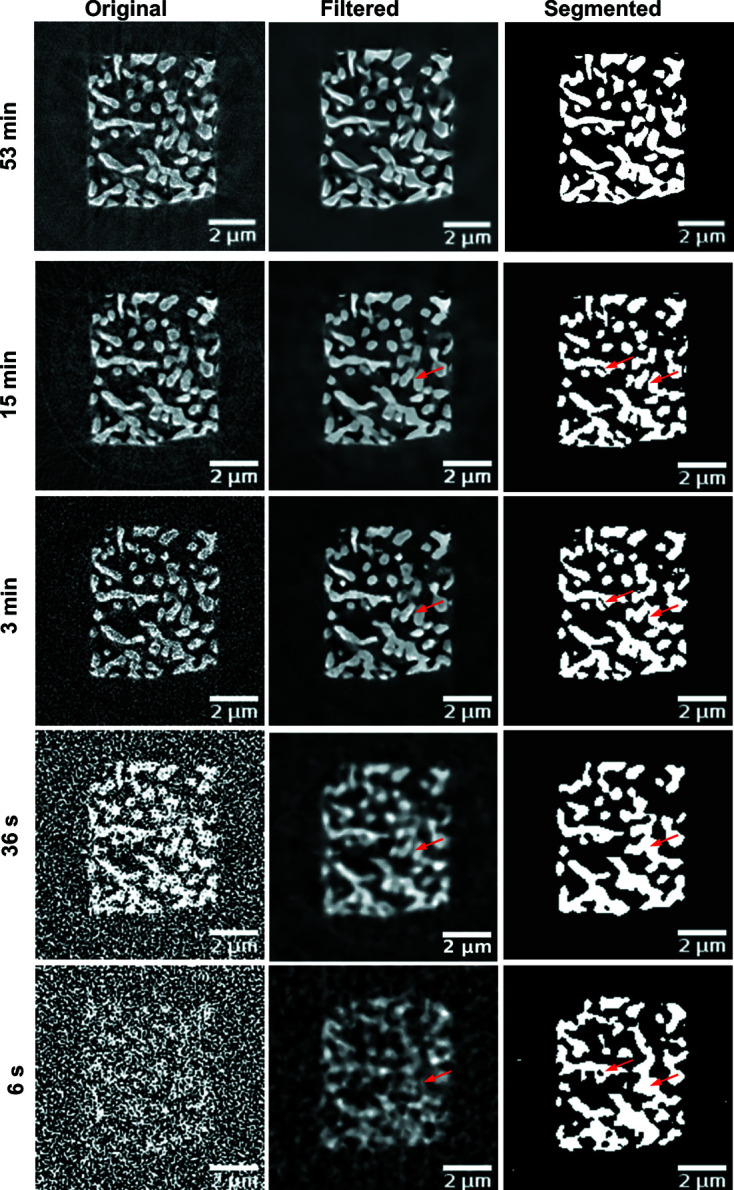
Reconstructed slices of NPG (absorption contrast) without any alignment or filtering (left). Slices after filters have been applied (middle): non-local means filter (53 min, 15 min), median 3D and non-local means (3 min), low-pass filter and non-local means (36 s), and low-pass filter and median 3D (6 s). Automated threshold segmentation of slices (right).

The image quality of the reconstructed slices strongly depends on the total exposure time (Fig. 2 ▸). In the 15 min scan, all features can be clearly resolved with high contrast. The 53 min scan is less noisy but artifacts from long-term sample movements can be recognized. In the 3 min scan, the features are clearly resolved and after applying filters the noise can be removed. In the 36 s scan the overall structure can be determined but the noise becomes a critical factor. Because of the high noise level, more filtering is needed so the filtered image looks more blurred and smaller details are lost (Fig. 2 ▸). In the unfiltered 6 s scan, no inner structures can be resolved, so intensive filtering is necessary. Nevertheless, the segmentation after filtering clearly shows similarities with the other segmentations and key features can be recognized.

Small features, like a gap of 150 nm in width between two individual ligaments indicated by the red arrows in Fig. 2 ▸, are resolved in the 15 min scan. Although the gap is clearly visible in the filtered 3 min image, the automated segmentation fails to resolve the gap completely.

### 3.2. Zernike phase-contrast X-ray microscope   

**Figure 3:**
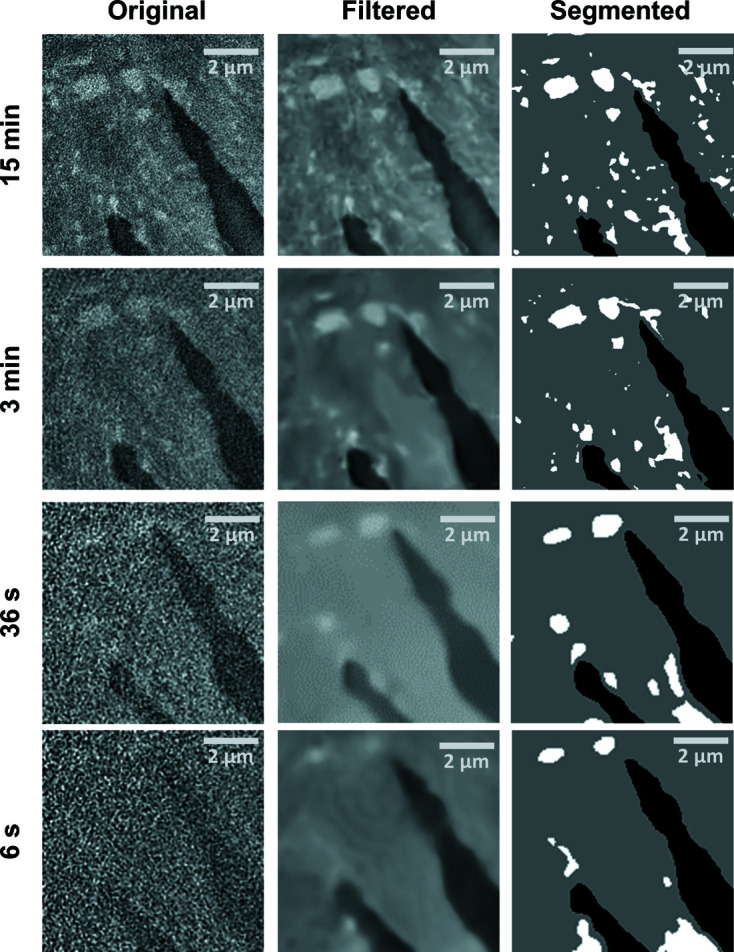
Reconstructed slices of magnesium (Zernike phase contrast) without any filtering (left). Filtering (middle) allows automatic segmentation (right). White spots depict SiC crystals, black areas indicate voids inside the material and grey areas depict the Mg matrix. Features of 150 nm size can be detected in the 15 min scan while already in the 3 min scan all features smaller than 300 nm are not extracted by the segmentation. Only rough structures are resolved in the 36 s and 6 s scans.

The inner structure of the low-absorbing magnesium composite can be determined using Zernike phase contrast (Fig. 3 ▸). In the 15 min scan, SiC particles of different sizes from 1.5 µm down to 150 nm, as well as voids inside the magnesium, are resolved. Because of the automated segmentation based on grey value thresholds, the segmentation of the 15 min scan does not represent the perfect sample structure but is used as a qualitative reference to compare the similarity of different scans. After filtering the 3 min scan, smaller particles (<300 nm) are no longer segmented correctly in the automated segmentation. In the short scans, the overall shape of the voids is clearly visible but only larger particles (>500 nm) are segmented correctly. In the 6 s scan, most of the information about the SiC particles is lost, and when using the automated segmentation the wrong particles are segmented. Nevertheless, the voids inside the magnesium are still resolved.

### 3.3. Contrast-to-noise ratio   

**Figure 4:**
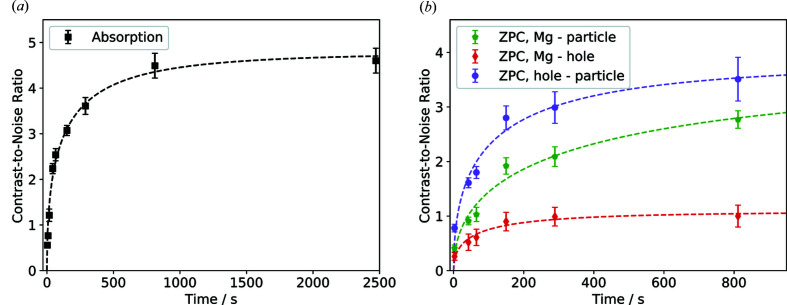
CNRs for different total exposure times *t* for absorption contrast (black) and Zernike phase contrast (colour). The CNR increases with 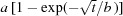 (the fit is shown as dotted lines). For more details about the fit see Table S3.

The calculated CNR increases, because of the Poisson photon statistics, with the number of photons and therefore with the total exposure time *t* (Fig. 4 ▸). The quality improves rapidly when scan times are increased and the effect levels off towards higher average count rates. On the other hand, sample- and optics-induced drifts can decrease the CNR, the resolution and the overall image quality of the reconstruction, when scan times are increased. The CNR does not only depend on the setup and scan time but also on the contrast of the sample itself.

The following function was fitted to the calculated CNR,
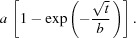
Here, *a* represents an upper limit of the achievable CNR while *b* describes how fast the CNR approaches the experimental limit. The maximum achievable CNR is limited. It is defined by the intrinsic material properties, like the variation in mean grey values and their standard deviation (Lovric *et al.*, 2013 ▸). Furthermore, the used instrument setup can limit the CNR, *e.g.* artifacts from sample movement caused by thermal drifts during long scan times.

For NPG in absorption, after 15 min a CNR of already 95% of the upper limit is reached, while it does not increase much with respect to the 53 min scan (97%). However, in the latter, artifacts, *e.g.* from long-term sample drift, can already be recognized and decrease the overall image quality (Fig. 2 ▸, upper left). At 3 min, it still has a CNR of 65% of the limit value. In this case, a good compromise between the CNR and short scan time for absorption is ∼15 min.

For the magnesium sample in Zernike phase contrast, the CNRs calculated for the different materials in the sample show different curve progressions. The highest CNR is recorded between SiC and air, which is expected because of the largest differences in β and δ. Here, 92% of the limit *a* is reached after 15 min. For the magnesium (CNRs for Mg–air and Mg–SiC), values of ∼75% of the CNR limit are reached after a scan time of 15 min. The halo effect observed in the Zernike reconstruction can lead to a wider range of grey values inside one material and therefore a larger σ, *i.e.* smaller CNR. The slower increase in CNR for the magnesium phases might be because of its heterogeneity in grey values (see also Fig. 3 ▸, upper row): regions of brighter and darker magnesium can be identified, suggesting that the SiC particles are not completely immersed in the magnesium phase. This suggests that an extension of the scan time towards 30 min could result in a further increase of the CNR and lead to better distinguishable magnesium phases.

### 3.4. Spatial resolution   

**Figure 5:**
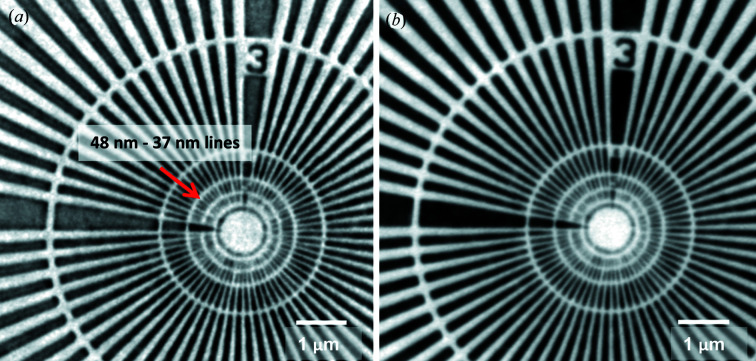
(*a*) Absorption contrast and (*b*) Zernike phase contrast of a Siemens star. The <50 nm lines (third inner ring, red arrow) are clearly resolved in both images. The half-period resolution (as determined by FRC) for (*a*) is 48.1 nm ± 1.6 nm and for (*b*) is 47.2 nm ± 2.5 nm.

**Figure 6:**
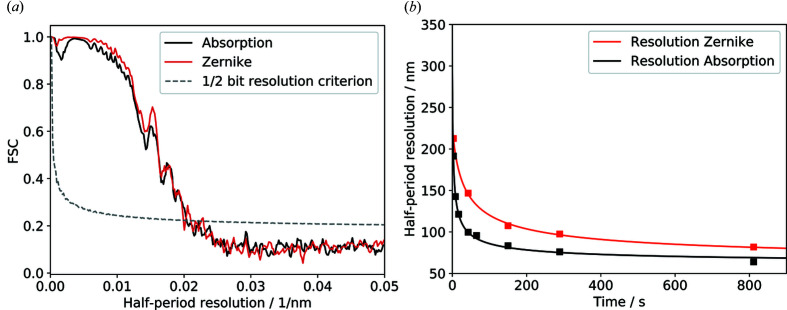
(*a*) Estimation of the resolution via FSC of a 2D Siemens star test pattern for absorption (black) and Zernike phase contrast (red). The best achieved half-period resolution in 2D is 47.2 nm ± 2.5 nm. (*b*) An overview of the calculated resolution for different scan times using the half-bit criterion. The best half-period resolution in 3D of 64.0 nm was achieved in the 15 min absorption scan using a binning of 2.

The resolution in 2D was estimated using a Siemens star test pattern with smallest feature sizes of 25 nm (Fig. 5 ▸). The lines of the third innermost ring (line widths between 48 nm and 37 nm) can clearly be resolved. The highest achievable half-period resolution estimated from the FRC using the half-bit resolution criterion is [Fig. 6 ▸(*a*)] 48.1 nm ± 1.6 nm for absorption and 47.2 nm ± 2.5 nm for Zernike phase contrast. This value is larger than the half-period optical resolution limit of the FZP of 30.5 nm (= 0.5 × 1.22 × 50 nm) corresponding to half of the Rayleigh criterion. This shows that the setup is limited by either the mechanics or the detector system. The calculated resolution values correspond to ∼3 pixels on the detector and are in line with the manufacturer specification (30 line-pairs mm^−1^ which equates to a half-period resolution of 2.5 pixels).

The resolution in 3D calculated for the absorption sample by the FSC improves when extending the scan times [Fig. 6 ▸(*b*)]; this is expected because the number of photons is proportional to the scan time, 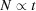. In this case, the 3D resolution does not reach the optical resolution limit as it is limited by noise. A half-period resolution of 64.0 nm ± 1.2 nm in 3D was achieved in the 15 min absorption scan with a binning of 2 and a resulting effective pixel size of 29.8 nm. A half-period resolution of 83.5 nm ± 1.6 nm was achieved for the 3 min scan. However, reducing the scan time further below 3 min leads to a significant decrease in spatial resolution. This is expected since the noise level is increasing because of the limited flux. In addition, the limited number of angles for the tomographic reconstruction decreases the reconstruction quality further. The spatial resolution in the Zernike phase contrast appears to be lower in the 3D volume (81.9 nm ± 1.6 nm for 15 min and 107.6 nm ± 4.3 nm for 3 min).

### 3.5. Improving image quality by machine learning   

**Figure 7:**
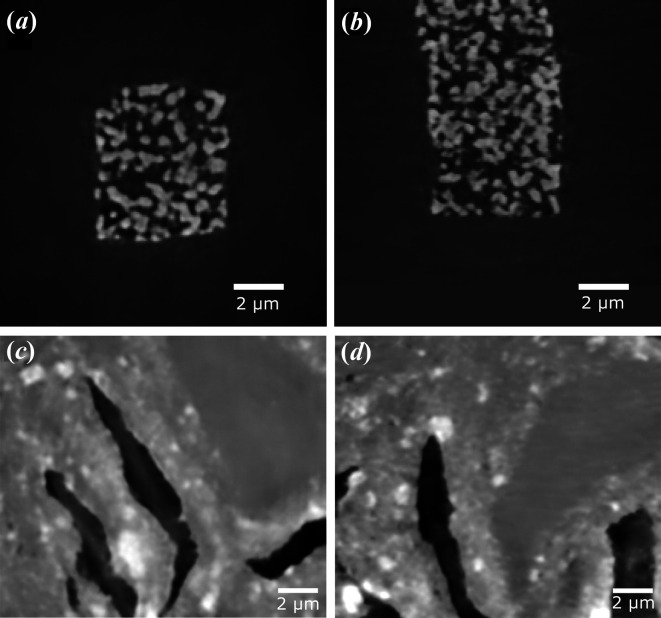
The result of training with the machine learning network *msdnet* (Pelt *et al.*, 2018 ▸; Pelt & Sethian, 2018 ▸) for short scans. (*a*) In the *x*–*y* direction and (*b*) in the *y*–*z* direction of a 6 s absorption scan of NPG. (*c*, *d*) Slices of a 36 s Zernike phase contrast scan of magnesium alloy in (*c*) the *x*–*y* direction and (*d*) the *x*–*z* direction. The noise is eliminated and the structures show great similarities to the structures observed in longer scans. For the original slice in the *x*–*y* direction, see Figs. 2 ▸ and 3 ▸, lower left.

A mixed-scale dense convolutional neural network (*msdnet*; Pelt *et al.*, 2018 ▸; Pelt & Sethian, 2018 ▸) was used to improve the image quality. The training was performed on 100 slices of the reconstructed 6 s scan (absorption) and 36 s scan (Zernike phase contrast), while the 15 min scan was used as a ground truth (target). The network was validated on 20 slices and tested on 20 slices (Fig. 7 ▸). Training the network on individual slices in the *x*–*y* direction leads to artifacts in the other direction (*z* direction). Therefore, for each slice the four closest adjacent slices were used as additional input channels, which eliminates the artifacts and improves the image quality in all directions. Fig. 7 ▸ shows that the noise is completely eliminated since the network only learns the ‘real’ structures and does not reproduce the random noise, so no additional noise-reduction filter is necessary. The structures of the NPG do match very well with the structures visible in Fig. 2 ▸. For the magnesium sample, the noise is reduced significantly, larger grain structures become clearer and smaller grains become visible. Altogether, machine learning is a very powerful alternative to classical filtering to reduce the noise and extract features in low-quality scans.

## 4. Discussion   

The ideal scan time of a specific sample in a nanotomography setup can be determined by analysing different parameters like CNR and spatial resolution. Using the nanotomography setup at P05, we can extract information about the samples reliably down to scan times of 3 min without loss of spatial resolution. Although the image quality of the unfiltered short scans appears to be very poor, filtering can enhance the image quality drastically so that even threshold segmentation is possible, but with some loss of detail.

A good compromise between the best CNR and short scan time is found to be at ∼15 min for both contrast methods. At 15 min, the CNR in the absorption scan is already close to the experimental limit and does not increase significantly when extending the scan time.

In the case of Zernike phase contrast, an increase in scan time might result in a further improvement of the CNR of this MgSiC material, in particular the CNRs of Mg–air and Mg–SiC. However, it has to be taken into account that at long scan times other factors, *e.g.* thermal drifts, become predominant and might lead to a reduction in the spatial resolution as well as the CNRs. For weakly absorbing biological samples, the dose becomes an important factor and therefore the scan times have to be considered more carefully. Here, a good compromise could be a 15 min scan with additional ML denoising. Improvements in spatial resolution are not expected for scan times longer than 15 min for both contrast methods.

For these two reference samples, the ideal scan time for the current setup at the P05 nanotomography endstation is ∼15–30 min for high-quality scans and 3 min for fast scans. Even though this is dependent on sample contrast and structure, these values can be used as a good guideline for other samples as well. The fast scan mode can, for example, be used when performing *in situ* experiments.

Another approach to profit from both high image quality and short scan times is to carry out a long high-quality scan before the *in situ* experiment and short scans during the *in situ* experiment. The image quality of the short time scans can then later be improved using machine learning (Yang *et al.*, 2018 ▸; Pelt *et al.*, 2018 ▸). First tests with *msdnet* showed that even for very short scan times the structure of the sample can be extracted after training the network using the 15 min scan as reference.

Time-resolved TXM with short scan times is feasible at the current setup of the nanotomography endstation at P05 for both absorption and Zernike phase contrast with high spatial resolution. This enables fast *in situ* experiments. It is to be expected that the overall scan time can be further decreased in the future, once the new double multilayer monochromator (DMM) at P05 is fully commissioned. The DMM can provide one order of magnitude more flux than the double-crystal monochromator used in this study.

Fast nanotomography using an X-ray microscope has the potential to bridge the gap between ultra-fast microtomography with time resolution down to a few milliseconds (García-Moreno *et al.*, 2019 ▸) and spatial resolution of down to 1 µm, and high-resolution imaging techniques such as focused ion-beam tomography and ptychography with spatial resolution down to a few tenths of a nanometre but several hours of acquisition time.

## Supplementary Material

Supporting figure and tables. DOI: 10.1107/S1600577520007407/mo5217sup1.pdf

## Figures and Tables

**Table 1:** Parameters of the different scan modes The scans highlighted in bold have only been measured in absorption. The exposure time has to be adapted so that blurring during the exposure is prevented. On the other hand, the efficiency decreases with more images because of the dead-time of the detector. A compromise between these two parameters has to be found. For faster scan times, smearing of more than one pixel was allowed to keep the efficiency reasonably high.

Total scan time	Total exposure time (s)	Exposure time (s)	Number of images	Binning	Efficiency
**53 min**	**2472**	**1**	**2472**	**1**	**0.77**
15 min	811.3	0.55	1475	2	0.90
6 min	288.9	0.22	1313	2	0.80
3 min	149.8	0.22	681	4	0.83
**1.5 min**	**65.0**	**0.11**	**591**	**4**	**0.72**
1 min	42.4	0.05	848	4	0.70
36 s	16.9	0.04	423	4	0.47
**18 s**	**8.1**	**0.033**	**245**	**4**	**0.44**
6 s	2.7	0.033	81	4	0.44
