# Supplementary material for: Pushing the temporal resolution in absorption and Zernike phase contrast nanotomography: enabling fast in situ experiments
Source: J Synchrotron Radiat. 2020 Jul 30;27(Pt 5):1339–46. doi: 10.1107/S1600577520007407 (PMC7467338; doi:10.1107/S1600577520007407)
Supplement: Supplementary file 1 [file s-27-01339-sup1.pdf]

JOURNAL OF SYNCHROTRON RADIATION

Volume 27 (2020)

Supporting information for article:

Pushing the temporal resolution in absorption and Zernike phase-contrast nanotomography: enabling fast in situ experiments

Silja Flenner, Malte Storm, Adam Kubec, Elena Longo, Florian Döring, Daniël M. Pelt, Christian David, Martin Müller and Imke Greving

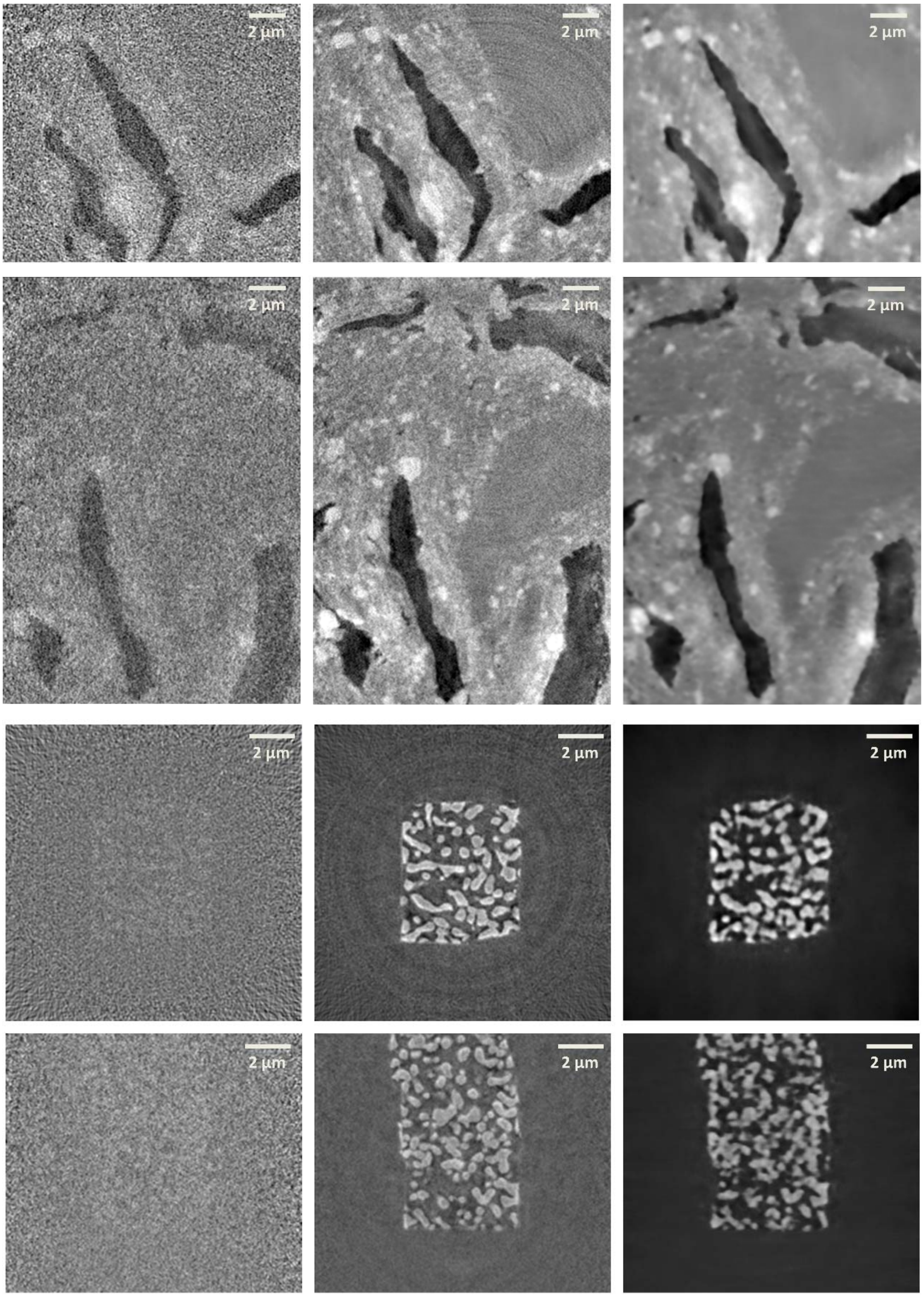

Figure S1 Upper row: input (left), target (middle) and network output of the machine learning with msdnet (Pelt *et al.*, 2018; Pelt & Sethian, 2018). The training was performed in the xy-plane. Lower row: xz- view of the corresponding dataset.

Table S1 Measured performance parameters of the air bearing rotation stage (Pi Micos), custom design.

Type of Error	Error σ
Axial error	16.92 nm
Radial error	21.43 nm
Tilt R_x	0.223 µrad
Tilt R_y	0.190 µrad

Table S2 Material parameter of the test samples. The real part of the refractive index δ describes a sample induced phase shift and β the extinction. β and δ are given for an energy of 11 keV.

Material	β [11keV]	δ [11keV]	Density g/cm3
NPG	$2.4\ 10^{-6}$	$1.5\ 10^{-5}$	19.3
Mg	$2.4\ 10^{-8}$	$2.9\ 10^{-6}$	1.7
SiC	$4.8\ 10^{-8}$	$5.46\ 10^{-6}$	3.2

Table S3 Parameters of the CNR fits $a\left(1-\exp\left(-\frac{\sqrt{t}}{b}\right)\right)$ displayed in Figure 4.

	a	b
NPG (absorption)	4.7	11.6
Mg – air	1.4	8.4
Si C– air	3.8	10.9
SiC – Mg	3.7	20.3
